# Development of an Expert-Based Scoring System for Early Identification of Patients with Inborn Errors of Immunity in Primary Care Settings – the PIDCAP Project

**DOI:** 10.1007/s10875-024-01825-3

**Published:** 2024-10-21

**Authors:** Jacques G. Rivière, Gerard Carot-Sans, Jordi Piera-Jiménez, Sergi de la Torre, PIDCAP expert group, Laia Alsina, Ana Mª Bielsa Masdeu, Maria Bosom Diumenjó, Javier Carbone, Carmen Carreras, Angela Deya-Martínez, Romina Dieli-Crimi, María Espiau, Luis Fernández Pereira, I. González, Manel Juan, Pilar LLobet, Andrea Martín-Nalda, Maria Mendez, Olaf Neth, J. Gonzalo Ocejo-Vinyals, Peter Olbrich, J. C. Rodríguez, Carmen Rodríguez-Vigil Iturrate, Carlos Rodrigo, Juan Luis Santos Pérez, Xavier Cos, Xavier Serra-Picamal, Pere Soler-Palacin

**Affiliations:** 1 Infection and Immunity in Pediatric Patients Research Group, Vall d’Hebron Institut de Recerca (VHIR), Barcelona, Catalonia Spain; 2 Pediatric Infectious Diseases and Immunodeficiencies Unit, Hospital Infantil I de La Dona Vall d’Hebron, Vall d’Hebron Barcelona Hospital Campus, Barcelona, Catalonia Spain; 3 Present Address: Universitat Autònoma de Barcelona (UAB), Barcelona, Catalonia Spain; 4 Jeffrey Modell Diagnostic and Research Center for Primary Immunodeficiencies, Barcelona, Catalonia Spain; 5 Catalan Health Service, Barcelona, Catalonia Spain; 6 Digitalization for the Sustainability of the Healthcare System (DS3) Research Group, L’Hospitalet de Llobregat, Catalonia Spain; 7 Faculty of Informatics, Multimedia and Telecommunications, Universitat Oberta de Catalunya, Barcelona, Spain; 8 Institut Català de La Salut (ICS), Barcelona, Catalonia Spain; 9 The Foundation University Institute for Primary Health Care Research Jordi Gol I Gurina (IDIAPJGol), Barcelona, Spain

**Keywords:** Inborn errors of immunity, primary immunodeficiencies, Jeffrey Modell Foundation, warning signs, rare disease, expert-driven scoring system, early diagnosis, computer-assisted medicine

## Abstract

Early diagnosis of inborn errors of immunity (IEIs) has been shown to reduce mortality, morbidity, and healthcare costs. The need for early diagnosis has led to the development of computational tools that trigger earlier clinical suspicion by physicians. Primary care professionals serve as the first line for improving early diagnosis. To this end, a computer-based tool (based on extended Jeffrey Modell Foundation (JMF) Warning Signs) was developed to assist physicians with diagnosis decisions for IEIs in the primary care setting. Two expert-guided scoring systems (one pediatric, one adult) were developed. IEI warning signs were identified and a panel of 36 experts reached a consensus on which signs to include and how they should be weighted. The resulting scoring system was tested against a retrospective registry of patients with confirmed IEI using primary care EHRs. A pilot study to assess the feasibility of implementation in primary care was conducted. The scoring system includes 27 warning signs for pediatric patients and 24 for adults, adding additional clinically relevant criteria established by expert consensus to the JMF Warning Signs. Cytopenias, ≥ 2 systemic infections, recurrent fever and bronchiectasis were the leading warning signs in children, as bronchiectasis, autoimmune diseases, cytopenias, and > 3 pneumonias were in adults. The PIDCAP (Primary Immune Deficiency “Centre d’Atenció Primària” that stands for Primary Care Center in Catalan) tool was implemented in the primary care workstation in a pilot area. The expert-based approach has the potential to lessen under-reporting and minimize diagnostic delays of IEIs. It can be seamlessly integrated into clinical primary care workstations.

**Supplementary Information:**

The online version contains supplementary material available at 10.1007/s10875-024-01825-3.

## Introduction

Inborn Errors of Immunity (IEIs), also known as primary immunodeficiencies (PI), are a category of diseases defined by the presence of a compromised immune system, mainly as a result of a single-gene loss-of-function or gain-of-function mutation. IEIs vary in the mechanism of action, mode of inheritance, prevalence, and severity. They have traditionally been characterized by an increase in susceptibility to severe and recurrent infections in the early years of life, with many patients having a poor prognosis with reduced quality of life and high rates of mortality [1]. However, patients with IEIs are now known to present with a wide range of presentations and an increasing number of clinical phenotypes. In addition to an increased frequency of infections, the development of immune dysregulation and autoimmunity, cancer, and allergy are also associated with IEIs [1–4].While individual IEIs may be rare, with as few as one or two cases reported in the literature, they collectively account for a significant burden of disease [5, 6].

Early diagnosis of IEIs has been shown to reduce rates of patient mortality and morbidity, healthcare costs, and improve quality of life for patients [6–10]. While the diagnostic delay of IEI after the first presentation of symptoms is decreasing, it is still typically between 1–4 years worldwide and 2–5 years within Europe, depending on the country and the IEI [11]. Low awareness of IEIs and lack of resources are among the primary causes of diagnostic delay [12–15]. The need for early diagnosis for IEIs has led to the development of tools that may trigger earlier clinical suspicion of IEIs by primary care physicians and pediatricians, which in turn could lead to earlier diagnosis and management. The most well-known of these tools is the SPIRIT analyzer which is based upon the 10 Warning Signs of Primary Immunodeficiency (JMF Warning Signs), created by the Jeffrey Modell Foundation in 1993 and updated twice since. The tool is based on a list of common warning signs aimed to help physicians identify individuals with a suspected IEI, available separately for pediatric and adult patients. The effectiveness of the JMF Warning Signs has often been evaluated regarding their ability to detect IEIs that present primarily as an increased susceptibility to infections but there are limitations among the broader range of IEI phenotypes [16–21]. Secondary warning sign lists have been produced in attempts to aid in the diagnosis of underlying IEIs in various indications, including lung disease, oncohematology, gastroenterology, dermatology, and infectious diseases, among others [2, 4, 22]. Previous attempts to revise the JMF Warning Signs in light of newly recognized warning signs have also been made, though most lists appear to be limited to a similar number and spectrum of total warning signs, with some efforts focusing on raising awareness primarily in the primary care setting [17]. More recently, attempts have been made to produce medical expert systems (computer tools to aid physicians in diagnosis and treatment choices) to facilitate the diagnosis of IEI within primary care settings [23–31]. These tools may offer a more modern solution to raise healthcare professionals’ awareness and clinical suspicion within a primary care setting. However, unlike highly prevalent diseases, which can be more easily investigated using retrospective data from electronic health records (EHRs), rare diseases such as IEIs are often underreported or are not properly registered. This feature challenges the development of predictive scoring systems based on statistical or machine-learning approaches, which require large amounts of high-quality data on both cases and controls.

With this in mind, we produced an expert-based scoring system, developed by both primary care physicians and immunologists in Spain, based on extended warning signs of the original 10 JMF Warning Signs, to be applied to primary care settings.

## Methods

### Study Overview and Setting

The PIDCAP project aimed to develop and implement a scoring system for the early identification of individuals with IEIs in the primary care setting. The project was led by a task force consisting of the following profiles: clinical experts and researchers from the Children’s Hospital at Vall d’Hebron Barcelona Hospital Campus (Catalonia, Spain), technical staff from the Catalan Institute of Health (ICS), primary care consultors, and a coordinator from *Innobics*—a virtual research platform allowing for the application and management of projects proposed by ICS professionals.

The PIDCAP scoring system was intended to be embedded into the clinical workstation of the Catalan Health Service’s primary care settings. The Catalan Health Service provides public, universal care to the entire population of Catalonia (8 million inhabitants) through a network of 64 general hospitals, 27 psychiatry hospitals, 375 primary care centers, 91 skilled nursing facilities for intermediate care, and 130 outpatient mental health facilities. All primary care centers of the Catalan Health Service share a single clinical workstation (i.e., the eCAP platform) and store all clinical information in a single clinical data repository. The ultimate goal of the PIDCAP scoring system was to incorporate a built-in alert system in the eCAP to identify patients at high risk of having an IEI and to subsequently trigger a referral to experts for further IEI investigation.

All data generated and used in this study were handled according to the General Data Protection Regulation 2016/679 on data protection and privacy for all individuals within the European Union and the local regulatory framework regarding data protection. Procedures were approved by the research ethics committee of the coordinating center PR(AMI)339/2017. By the time of admission for specialized assessment, all historical clinical data of the patients, irrespective of the center they regularly visited, was requested and included in the EHR of the admitting hospital.

### Scoring System Development

Two scoring systems were conceived: one for the pediatric population (i.e., younger than 14 years, as defined by the Catalan Health Service) and one for the adult population (i.e. 14 years of age and older). We used a qualitative Delphi methodology for developing the scoring systems that would be utilized by the PIDCAP tool. The entire process consisted of three phases: (1) identification of warning signs of IEI and preliminary significance weighting, (2) expert consensus on the inclusion and weighting of each sign within the scoring systems, and (3) assignment of structured disease codes to the identified warning signs.

Potential warning signs known to suggest the presence of an IEI in both adult and pediatric patients were determined via an in-depth literature review. PubMed and Google Scholar databases were screened for English-language articles reporting studies to identify risk factors for IEI, including those developing and/or validating scoring system to this end. The search combined key terms (including variants with the same root terms) regarding primary immunodeficiencies and warning signs or screening strategies. Searches were restricted from 1993 onward based on the first published list of JMF warning signs, with no further restrictions. The search strategy and result are described in the Supplementary Material. The task force members reviewed all articles and extracted the risk factors, then collaborated with 8 local experts (consisting of 3 pediatric immunologists, 1 primary care pediatrician, 3 adult immunologists, and 1 primary care general practitioner) to come up with an initial list of warning signs and their weighting within the scoring system, by means of a score between 10 and 75. Cut-off values were arbitrarily established: high risk (score ≥ 75), moderate risk (35–70), and low risk (< 35).

For the second phase, we set up a panel of experts to rate the relevance of each of the pre-identified warning signs and its relative contribution (i.e., weight). We selected 36 experts from across reference centers in Catalonia and Spain attending to pediatric and/or adult patients with IEIs, and primary care physicians for their expertise and realistic view of primary care pitfalls. Candidates were invited to participate via email questionnaire with a response deadline set 5 weeks following invitation. Accompanying the questionnaire, the enrolled experts also received a summary of the chosen literature, as well as information on the rationale and background of the PIDCAP project for context. Experts were asked to rate the inclusion and the suggested weighting for each warning sign on a 1-to-4 scale based on their relative perceived significance in the clinical suspicion of an underlying IEI. Experts could also suggest alternative weightings.

Consensus was considered when a given sign/weight scored 3 or higher by at least half of the participating experts. In cases in which consensus for the suggested weighting of a warning sign was not reached, they were reevaluated by the local task force based on expert feedback. Items not reaching consensus could be either removed or redefined. The final adult and pediatric scoring systems were sent to the experts, along with the results of the consensus process; the experts were offered the opportunity to suggest final amendments if deemed necessary.

Finally, the local task force translated the list of warning signs (typically reported in the literature in natural language) into codes of the International Classification of Diseases version 10, Clinical Modification (ICD-10-CM).

### Retrospective Testing in the IEI Cohort

The resulting scoring system was tested against a retrospective registry of patients with confirmed IEI at the coordinating center, using data from their primary care EHR. The Vall d’Hebron Barcelona Hospital Campus is recognized as a reference center regionally and nationally and it is one of the 3 recognized European Reference Network for Rare Immunological Disorders (ERN-RITA) centers for IEI in Spain. The hospital serves more than 950,000 patients per year referred from local primary health care (with a catchment population of more than 500,000 individuals), but also referred from all over Spain (especially in rare diseases).

Pediatric and adult patients diagnosed with an IEI and registered in the hospital database between April 2005 and January 2023 were included in the analysis dataset. Data on previous diagnoses were cross-tabulated with the healthcare registry for diagnoses of the Catalan Ministry of Health, which collects all diagnoses reported to the SISAP central registry for primary care. The following individuals were excluded: pediatric patients with selective IgA deficiency, usually asymptomatic; adult individuals with less than 5 diagnosis entries in the primary care registry (corresponding to p10 of the number of adult diagnosis distribution) were considered non-representative on the assumption that less than 5 diagnosis entries meant that there were not followed within the public, universal primary care system but rather followed by either a private healthcare system or outside of Catalonia.

All patients in the analysis dataset were evaluated using the scoring system, based on their history of diagnoses recorded in the primary care registry before IEI diagnosis. For the retrospective test, the warning sign of ‘confirmed IEI’, introduced to the scoring system to ensure adequate follow-up of individuals with previous IEI diagnosis, was removed as all individuals in the registry already met the criteria. We estimated the number and percentage of individuals allocated by the scoring system in each of the risk categories and the frequency and percentage of each of the warning signs. The analysis consisted of descriptive statistics, and no hypothesis testing was conducted.

### Pilot Implementation in Routine Care

In April 2018, a pilot study was initiated to test the feasibility of using the PIDCAP scoring system in a clinical workstation of a primary care team. The scoring system was made available to all healthcare professionals of the *El Carmel*, Barcelona, primary care team, which provides care to a catchment population of 19,391 individuals: 16,794 adults and 2,597 children. The scoring system activated an alarm along with a referral recommendation for an expert evaluation for all patients identified at high risk of IEI. All referrals were recorded and communicated to the PIDCAP taskforce.

In addition to the PIDCAP scoring system, a series of educational sessions in primary and tertiary care centers was carried out between October 2017 and January 2018 to prepare other locations receiving the PIDCAP scoring system and to raise awareness and clinical suspicion of IEIs within these locations. Also, a series of infographics were produced to highlight the potential warning signs of IEIs in different indications. Dissemination of these resources was successfully achieved throughout the target area in line with the goals of the project.

## Results

### Scoring System Development

Warning signs for consideration were extracted from the literature search reporting warning signs and risk factors for primary immunodeficiency, and from medical experience of the task force team. The literature review, followed by collaboration with local experts, yielded 28 warning signs for the pediatric list and 22 for the adult list.

Of the surveyed panel of 36 experts, 22 (61%) answered the online survey: 16 the questionnaire for pediatrics and 10 for adults. The general characteristics of the experts are summarized in Table S1.

**Fig. 1:**
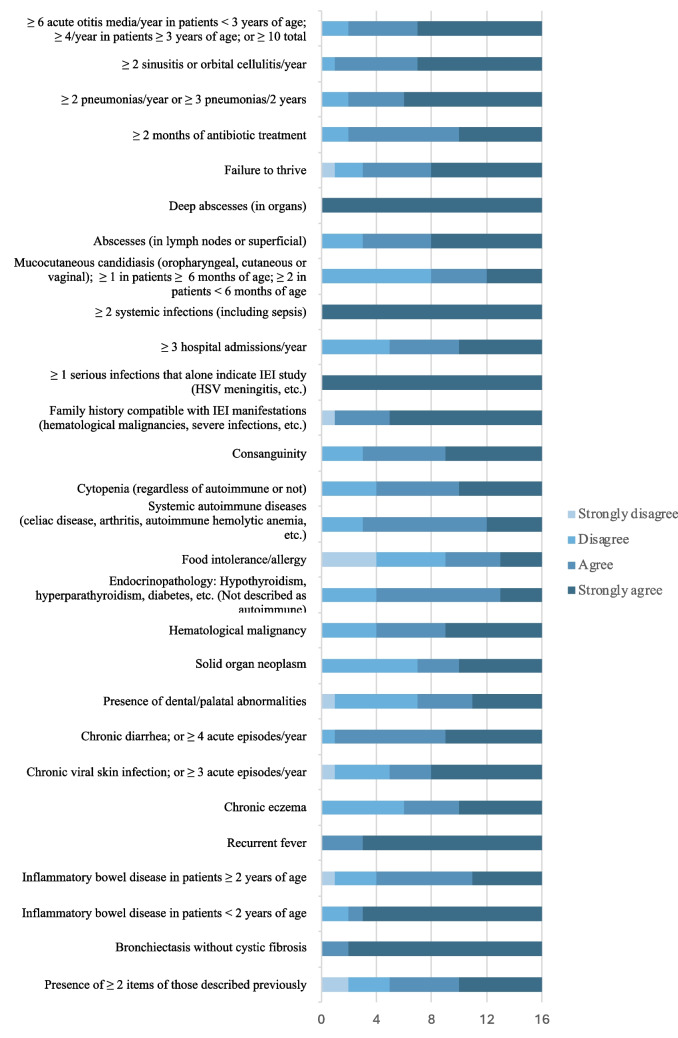
Agreement refers to the degree of agreement or disagreement with the proposed items and their weight. Results from 16 survey respondents, 11 of whom are identified as pediatric immunologists, 1 as an adult/general immunologist and 4 as primary care pediatricians. HSV: Herpes Simplex Virus, IEI: inborn errors of immunity

Of the 28 warning signs originally included in the survey for pediatrics, 27 (96%) reached the pre-established consensus threshold (Fig. 1); food allergy did not achieve a minimum of 50% agreement or strong agreement. Average score ranged from 2.8 to 4.0 (Table S2). After taking into account the expert review, one warning sign (‘food allergy’) was removed, and one item (‘presence of cytopenia’) was further separated into separate warning signs (‘cytopenia (regardless of autoimmune or not)’ and ‘autoimmune cytopenia’) with two different weightings. Furthermore, two further items were not used later due to limitations in primary care coding (‘ ≥ 3 hospital admissions/year’ and ‘ ≥ 2 months of antibiotic treatment’). The weighting of ten items was adjusted based on low score, free comments and coding limitations. One item (‘vaccine reaction’) was added according to expert recommendation, validated by the local task force. Table 1 shows the final scoring system for the pediatric population, which includes 27 warning signs.

**Table 1:** Warning signs included in the final pediatric scoring system

Warning sign	Weight in scoring system	Warning sign	Weight in scoring system
Pediatric patients			
≥ 10 acute otitis media^a^	20	Systemic autoimmune diseases, not including autoimmune cytopenia (celiac disease, arthritis, etc.)	30
≥ 3 sinusitis or orbital cellulitis^a^	20	Endocrinopathology: Hypothyroidism, hyperparathyroidism, diabetes, etc. (Not described as autoimmune)	30
≥ 3 pneumonia^a^	40	Hematological malignancy	30
Failure to thrive	20	Solid organ neoplasia (only those that have been associated with inborn errors of immunity in pediatrics: thyroid)	30
Deep abscesses (in organs)	75	Oral (dental/palatal) anomalies	20
≥ 3 recurrent skin abscess	20	Chronic diarrhea; or ≥ 10 episodes of acute diarrhea)^a^	30
Mucocutaneous candidiasis (oropharynx, cutaneous, excluded vaginal) in patients ≥ 12 months of age: ≥ 2 episodes^a^	30	Chronic viral skin infection; or ≥ 20 acute episodes	10
≥ 2 systemic infections (including sepsis)	75	Chronic eczema or other dermatological manifestations related to inborn errors of immunity	10
≥ 1 serious infections that alone indicate IEI study (meningitis caused by HSV, etc.)	75	Recurrent fever	75
Family history of inborn errors of immunity^a^	50	Inflammatory bowel disease in patients ≥ 2 years of age	30
Consanguinity or other family history compatible with manifestations of inborn errors of immunity (lymphomas, etc.)^a^	30	Inflammatory bowel disease in patients < 2 years of age	75
Cytopenia (not specified as autoimmune)	20	Bronchiectasis without cystic fibrosis	75
Autoimmune cytopenia^a^	40	Vaccine reaction^b^	20
Presence of 2 or more warning signs	10		

^a^Warning sign criteria amended following the results of the survey based on additional feedback provided by the surveyed experts, and to align with real-world implementation via coding

^b^Warning sign added based on recommendation from surveyed expert, later reviewed and implemented by local task force

Warning sign for ‘Food intolerance/allergy’ removed following the result of the survey. Warning signs for ‘2 or more months of antibiotic treatment’ and ‘3 or more hospital admissions/year’ removed due to limitations in ICD-10-CM coding within the electronic health record system

**Fig. 2:**
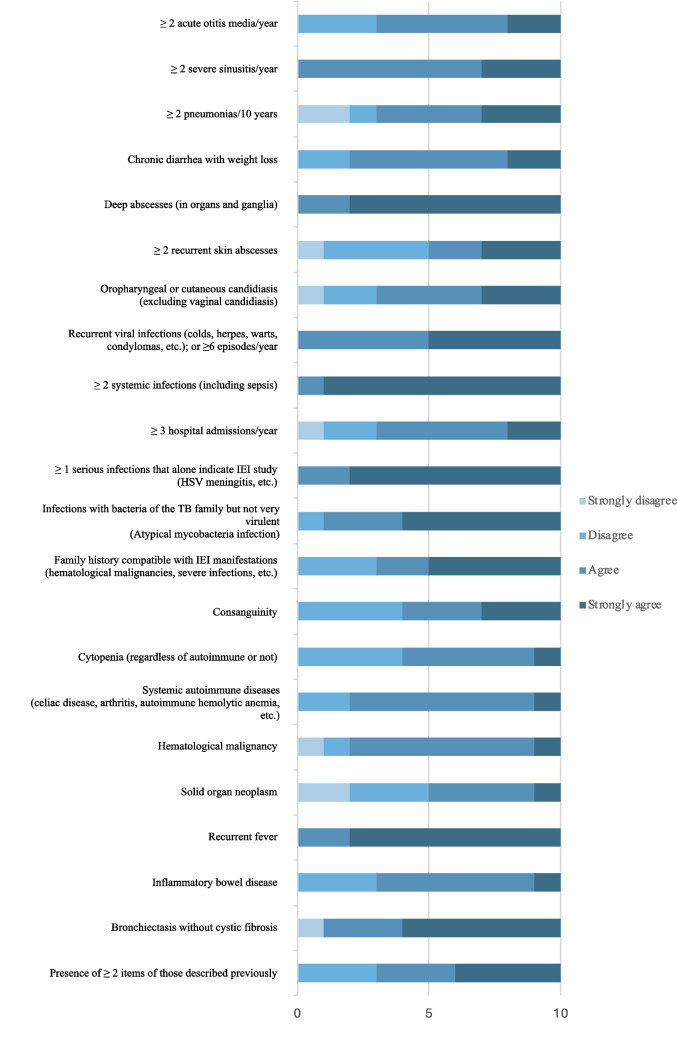
Agreement refers to the degree of agreement or disagreement with the proposed items and their weighting. Results from 10 survey respondents, 9 of whom are identified as adult immunologists, and 1 as a primary care physician. HSV: Herpes Simplex Virus, IEI: Inborn errors of immunity, TB: Tuberculosis

Regarding the adult survey, all 22 warning signs reached the consensus threshold (Fig. 2); the average score ranged from 2.9 to 3.9 (Table S3). One item was partitioned into two (as above, ‘presence of cytopenia’). Likewise, one item was not used due to coding limitations (‘ ≥ 3 hospital admissions/year’), the weighting of 8 items was adjusted based on low score, free comments and coding limitations, and two items (‘Oral (dental/palatal) anomalies’ and ‘chronic eczema or other dermatological manifestations related to inborn errors of immunity’) were added according to expert recommendation, and later validated. Table 2 shows the final scoring system for adults, which includes 24 warning signs.

**Table 2:** Warning signs included in the final adult scoring system

Warning sign	Weight in scoring system	Warning sign	Weight in scoring system
Adult patients			
≥ 8 acute otitis media^a^	30	Consanguinity or other family history compatible with manifestations of inborn errors of immunity (haematological neoplasms)^a^	30
≥ 8 sinusitis or chronic sinusitis^a^	30	Presence of cytopenia (without specifying if autoimmune)	20
≥ 3 pneumonia^a^	30	Autoimmune cytopenia	40
Chronic diarrhea	30	Presence of bronchiectasis without cystic fibrosis	75
Deep abscesses (in organs and/or ganglia)	50	Systemic and endocrine autoimmune diseases (celiac disease, arthritis, systemic lupus, thyroiditis, etc.)	30
Recurrent skin abscesses of repetition (3 or more)^a^	20	Haematological neoplasia (excluding multiple myeloma, chronic myeloid leukemia, Waldenström’s disease, etc.)^a^	50
Oropharyngeal or cutaneous candidiasis (excluding vaginal candidiasis)	30	Solid organ neoplasia (only those related with inborn errors of immunity: skin, stomach, thyroid)^a^	30
Recurrent viral infections (colds, herpes, warts, condylomas, etc.) 25 or more episodes^a^	30	Inflammatory bowel disease	20
2 or more systemic infections including sepsis	75	Recurrent fever	50
Unique severe condition that alone require study for inborn errors of immunity	75	Oral (dental/palatal) anomalies^b^	20
Atypical mycobacteria infection	50	Chronic eczema or other dermatological manifestations related to inborn errors of immunity^b^	10
Family history of inborn errors of immunity^a^	50		
Presence of 2 or more warning signs	10		

^a^Warning sign criteria amended following the results of the survey based on additional feedback provided by the surveyed experts, and to align with real-world implementation via coding

^b^Warning sign added based on recommendation from surveyed experts, later reviewed and implemented by local task force. Warning signs for ‘3 or more hospital admissions/year’ removed due to limitations in ICD-10-CM coding within the electronic health record System

*HSV* Herpes Simplex Virus

Of the total 68,000 possible ICD-10-CM codes reviewed following the survey, 3,387 for pediatrics and 3,497 for adults were retained and assigned to each warning sign (See Supplementary Material for the ICD-10-CM codes). Subsequently, the task force adapted the scoring system to the specifications of the eCAP clinical computer system of the Catalan Health Service, taking into account the changes introduced after the survey. Each warning sign was paired manually by the task force with ICD codes as specified earlier. In routine care, each visit to the primary care center is considered an episode by the system, and coding with ICD is mandatory. Some WS were assigned a number of episodes to overcome intrinsic limitations of the coding system (e.g., chronic diarrhea was decided to be equivalent to 10 acute episodes).

### Retrospective Testing in an IEI Cohort

Overall, 305 patients (184 children and 121 adults) within the Vall d’Hebron Barcelona Hospital Campus records met the selection criteria for IEI and were, therefore, included in the study. The main demographic characteristics of the patients included in the study are summarized in Table S4 and the diagnosis group classification in Tables S5 and S6.

The scoring system classified 60 (32%) pediatric patients with confirmed IEI as high-risk individuals (Table 3); 47 (78%) had 2 or more warning signs. Chronic eczema, cytopenias, two or more systemic infections, recurrent fever, failure to thrive, and bronchiectasis in absence of cystic fibrosis were the leading warning signs. However, chronic eczema and failure to thrive were also present in individual children identified as low-risk by the scoring system.

**Table 3:** The scoring system classifications of pediatric patients with confirmed IEI

Warning signs	Risk			Total
	High	Medium	Low	
Pediatric IEI cohort retrospective test				
≥ 10 acute otitis media	3	1	0	4
≥ 3 sinusitis or orbital cellulitis	0	0	0	0
≥ 3 pneumonias	8	3	0	11
Failure to thrive	12	5	7	24
Deep abscesses (in organs)	0	0	0	0
≥ 3 recurrent skin abscesses	11	5	2	18
Mucocutaneous candidiasis (oropharynx, cutaneous, excluded vaginal) in patients ≥ 12 months of age: ≥ 2 episodes	2	0	0	2
≥ 2 systemic infections (including sepsis)	17	0	0	17
≥ 1 serious infection that alone indicate IEI study (Meningitis caused by HSV, etc.)	10	0	0	10
Family history of inborn errors of immunity	1	1	0	2
Consanguinity or other family history compatible with manifestations of inborn errors of immunity (lymphomas, etc.)	4	3	0	7
Cytopenia (not specified as autoimmune)	24	8	6	38
Autoimmune cytopenia	5	1	0	6
Systemic autoimmune diseases, not including autoimmune cytopenia (celiac disease, arthritis, etc.)	11	2	2	15
Endocrinopathology: Hypothyroidism, hyperparathyroidism, diabetes, etc. (Not described as autoimmune)	7	1	0	8
Hematological malignancy	2	1	1	4
Solid organ neoplasia (only those that have been associated with inborn errors of immunity in pediatrics: thyroid)	0	0	0	0
Oral (dental/palatal) anomalies	11	1	5	17
Chronic diarrhea; or ≥ 10 episodes of acute diarrhea)	2	0	1	3
Chronic viral skin infection; or ≥ 20 acute episodes	0	0	0	0
Chronic eczema or other dermatological manifestations related to inborn errors of immunity	22	6	14	42
Recurrent fever	14	0	0	14
Inflammatory bowel disease in patients ≥ 2 years of age	0	0	0	0
Inflammatory bowel disease in patients < 2 years of age	0	0	0	0
Bronchiectasis without cystic fibrosis	12	0	0	12
Vaccine reaction	0	0	0	0
Presence of 2 or more items of those described above	47	16	0	63
Number of patients	60	20	104	184

The scoring system classified 36 (30%) adult patients with confirmed IEI as high-risk individuals (Table 4). Nearly all of them (33/36 (92%)) had 2 or more warning signs. The most frequent warning sign among adults identified as high risk was bronchiectasi in the absence of cystic fibrosis, followed by systemic and endocrine autoimmune diseases, cytopenias, and more than 3 pneumonias. Of note, systemic and endocrine autoimmune diseases were also present in individual adults identified as low-risk by the scoring system.

**Table 4:** The scoring system classifications of adult patients with confirmed IEI

Warning Signs	Risk			Total
	High	Medium	Low	
Adult IEI cohort retrospective test				
≥ 8 acute otitis media	0	0	0	0
≥ 8 sinusitis or chronic sinusitis	0	0	0	0
≥ 3 pneumonias	10	0	1	11
Chronic diarrhea	5	0	1	6
Deep abscesses (in organs and/or ganglia)	1	0	0	1
Recurrent skin abscesses of repetition (3 or more)	0	0	0	0
Oropharyngeal or cutaneous candidiasis (excluding vaginal candidiasis)	8	2	0	10
Recurrent viral infections (colds, herpes, warts, condylomas, etc.) 25 or more episodes	1	0	0	1
2 or more systemic infections including sepsis	8	0	0	8
Unique severe condition that alone require study for inborn errors of immunity	6	0	0	6
Atypical mycobacteria infection	1	0	0	1
Family history of inborn errors of immunity	1	0	0	1
Consanguinity or other family history compatible with manifestations of inborn errors of immunity (hematological neoplasms)	1	0	0	1
Presence of cytopenia (without specifying if autoimmune)	17	5	2	24
Presence of bronchiectasis without cystic fibrosis	20	0	0	20
Autoimmune cytopenia	4	3	0	7
Systemic and endocrine autoimmune diseases (celiac disease, arthritis, systemic lupus, thyroiditis, etc.)	18	7	13	38
Hematological neoplasia (excluding multiple myeloma, chronic myeloid leukemia, Waldenström’s disease, etc.)	8	1	0	9
Solid organ neoplasia (only those related with inborn errors of immunity: skin, stomach, thyroid)	1	1	1	3
Inflammatory bowel disease	2	0	1	3
Oral (dental/palatal) anomalies	4	1	3	8
Chronic eczema or other dermatological manifestations related to inborn errors of immunity	1	2	1	4
Recurrent fever	1	0	0	1
Presence of 2 or more items of those described above	33	9	0	42
Number of patients	36	13	72	121

### Pilot Implementation

During the pilot implementation period, the PIDCAP scoring system tested 16,794 adults and 2,597 children; of them, 286 (1.8%) adults and 13 (0.5%) children were identified as high risk for IEI. Table S7 summarizes the main demographic characteristics of the source population. Primary care physicians were notified to follow up with these patients. An alert in each patient’s EHR was displayed to the primary care physician. These alerts specifically outlined the ICD-10-CM codes considered, the patient’s overall score, and extracted the most recent blood count along with any recorded immunoglobulin levels. Within this alert system, healthcare professionals were presented with three actionable options: (1) request a fundamental immunological work-up (inclusive of a full blood count and immunoglobulin levels) and wait for the results to determine the next step, (2) virtually refer the patient to a specialized reference center, or (3) arrange an in-person referral visit to the reference center. Additionally, primary care professionals had the discretion to dismiss the alert if they deemed that referral unnecessary due to an alternative explanation or if the patient was already being followed up. The workflow was very well received in primary care centers. After primary care assessment, a total of 40 adult and 3 pediatric patients were referred for further immunological evaluation. Unfortunately, owing to the prioritization criteria during the global COVID-19 healthcare crisis, no further follow-up information is available.

## Discussion

In this study, we developed an expert-based scoring system for identifying individuals with IEI based on diagnoses recorded in the primary care setting. The resulting scoring system expanded on the 10 classical warning signs considered for IEI screening with additional clinically meaningful items. The scoring system, which can be effectively implemented in primary care workstations, showed the ability to identify individuals at high-risk of IEI using retrospective data stored in primary care records.

The development of scoring systems or algorithms for automatic and early identification of individuals at high risk of IEI in the primary care setting faces two important challenges to be considered. First, the very low number of diagnoses hinders the development of sophisticated statistical models to predict the presence of IEI with adequate accuracy [32]. Second, the scarcity of screenings, including genetic investigations on individuals with clinical features suggestive of IEI has also been associated with high levels of underreporting [15, 33, 34]. Thus, some individuals considered to be controls in this scoring system development might be undiagnosed cases.

Despite these challenges, several authors have worked to expand the 10 JMF Warning Signs [6, 18, 34] mostly by including non-infectious comorbidities, under-represented in the JMF list, such as autoimmune disorders [18], or hematological signs [6], among others. Likewise, the panel of experts who participated in the PIDCAP study, identified non-infectious conditions, such as cytopenia, systemic autoimmune diseases, and chronic skin conditions, which may raise suspicion of an IEI and may be important warning signs of IEI. These findings also aligned with previous work by Dąbrowska et al., who highlighted the importance of other warning signs (hematooncologics, autoimmunity, and eczema) with similar findings [35].

The application of our scoring system should be considered within its intended use, which is automatically assessing the risk of IEI from information stored in primary care EHRs. This approach precludes warning signs that are not adequately reported or reliable in primary care records. This is the case for family history of IEI, which has been highlighted as an important predictor of IEI [16, 21, 35–37]. While these indicators can be assessed during a clinical interview based on a suspicion of immune disorder, they are not systematically reported in EHRs; therefore, their inclusion in a scoring system intended to automatically retrieve the IEI based on primary care records has limited utility. Another feature of the primary care setting that needs attention is the accuracy of some diagnoses. For instance, in our settings, the highly regulated prescription system in primary care requires a record of confirmed bacterial infection before prescribing antibiotics due to suggestive symptoms of pneumonia or ear infection. Therefore, these two conditions were intentionally underscored in our scoring system. These nuances, discernable only by primary care physicians, highlight the importance of including such healthcare professionals in a panel of experts responsible for developing automated scoring systems and algorithms for screening IEI risk in the overall population.

Another important challenge of this strategy is balancing sensitivity and specificity effectively. Unlike highly prevalent conditions, in which adequate balance between the false-positive and false-negative rates is desirable, the low prevalence of IEI indicates that even low false-positive rates would result in a many individuals unnecessarily referred to specialized services. This could overwhelm the healthcare system and result in a loss of confidence in the screening method. Our retrospective test using real-world data showed that the scoring system, with the pre-established threshold for positivity, would have missed 70% and 68% of adult and pediatric cases, respectively. However, considering the trade-off between specificity and sensitivity, we believe that this performance would increase the detection rate of IEI without an excessive number of false-positive cases unnecessarily referred to specialized services. Although some cases may go undetected, identifying cases that would otherwise be unnoticed without overloading hospital services is expected to produce important savings for the healthcare system [10].

Our scoring system showed poorer performance in identifying individuals at high risk of IEI than previous studies. However, such scoring systems typically require expertise or more sophisticated tools for extracting warning signs from EHRs [18, 29–31, 37, 38]. Our approach resulted in an interpretable scoring system that can readily extract and utilize diagnoses routinely recorded in the EHR. Another strength of this approach is the applicability across all age groups of individuals to be screened.

While these advantages and the results obtained so far encourage further development of the scoring system, our approach and analysis have some limitations that need to be considered. First, as with all assessments of rare conditions, our scoring system could not be tested on a sample large enough to validate its performance fully. Such validation is unlikely to be possible in a healthcare system like Catalonia with 8 million people. Rather, it will likely require federated learning approaches with multiple countries and benchmarking to similar scoring systems like those published by Messelink et al. [29], or studies utilizing the JMF SPIRIT analyzer in conjunction with other methodologies as published by Rider et al. [28, 30, 31] as emphasized in the AIPID 2023 workshop [39]. The initial step to mitigate this limitation will be to conduct an international Delphi consensus with the support of patient advocacy groups and scientific societies. Second, we encountered some technical limitations regarding the integration of drug prescriptions into the scoring system, which could significantly enhance its value. This issue is now being addressed in an upcoming version. Third, for this scoring system to be applied, primary care data must be collected and stored at the population level. Although healthcare increasingly relies on data, this level of data integration in the primary care setting is still uncommon. Finally, the COVID-19 crisis interrupted the pilot project to integrate the scoring system into the workstation of healthcare professionals, resulting in two important consequences: a relatively limited number of individuals screened (16,794 adults and 2,597 children) and the inability to follow up on and comprehensively characterize all cases identified by the scoring system.

The initial stages of the PIDCAP project show the usefulness and feasibility of an automated expert-based scoring system with expanded warning signs for identifying individuals at high risk of IEI among the general population using previous diagnoses reported in EHRs. While limited in overall performance, our model can contribute to reducing under-reporting and delayed diagnoses in individuals with IEI at a low cost and without overwhelming the healthcare system. Subsequent work in this regard should include standardizing the WS definitions so that they can fit into the clinical information structure of electronic health records from different countries. Envisioning a future scenario, the rapid advancement of large language models paves the way for enriching ICD codes with structured information extracted from medical histories using natural language processing. Owing to the scarcity of IEI cases overall, data federation approaches will be necessary to move forward in the development and validation of data-driven models for IEI screening.

## Supplementary Information

Below is the link to the electronic supplementary material.

Supplementary file1 (PDF 556 KB)

## Data Availability

No datasets were generated or analysed during the current study.
