# Supplementary material for: Development of an Expert-Based Scoring System for Early Identification of Patients with Inborn Errors of Immunity in Primary Care Settings – the PIDCAP Project
Source: J Clin Immunol. 2024 Oct 21;45(1):26. doi: 10.1007/s10875-024-01825-3 (PMC11493793; doi:10.1007/s10875-024-01825-3)
Supplement: Supplementary file 1 — Supplementary file1 (PDF 556 KB) [file 10875_2024_1825_MOESM1_ESM.pdf]

Development of an expert-based scoring system for early identification of patients with inborn errors of immunity in primary care settings – the PIDCAP project

Supplementary file

Contents

Literature search strategy and result 2

Methods 2

Search result 2

Table S1. Survey response distribution among healthcare providers 3

Table S2. Survey results for pediatric warning signs 4

Table S3. Survey results for adult warning signs 5

List of ICD-10-CM codes associated with each warning sign 6

Table S4. Main demographic characteristics of patients included in the retrospective testing of the scoring system in a cohort of patients with inborn errors of immunity. No. (%) 12

Table S5. International Union of Immunological Societies (IUIS) diagnosis group classification of pediatric patients included in the retrospective testing of the scoring system in a cohort of patients with inborn errors of immunity. 13

Table S6. International Union of Immunological Societies (IUIS) diagnosis group classification of adult patients included in the retrospective testing of the scoring system in a cohort of patients with inborn errors of immunity. 14

Table S7. Main demographic characteristics of the source population for the pilot implementation. No. (%) 15

Literature search strategy and result

Methods

Search purpose and strategy overview

The search was aimed at identifying relevant articles reporting information on clinical features, including warning signs for the screening of inborn errors of immunity.

Search strings were prepared to cover the following concepts: ‘warning signs’, ‘JMF warning signs’, ‘PID screening’, ‘primary immune deficiency diagnosis’, ‘computer-based algorithm immunodeficiencies’, ‘non-infectious manifestations of primary immune deficiencies’, ‘cancer in primary immune deficiencies’, and ‘autoimmunity in primary immune deficiencies’

To avoid excessiveness of non-relevant articles addressing the topic indirectly, search terms were to be either in the title or abstract when possible.

Selection criteria

We included peer-reviewed articles written in English and published between January 1993 and January 2018 addressing clinical manifestation or sign-based screening strategies for primary immune deficiencies.

Search strings

- **PubMed:** (("primary immunodeficien*"[Title/Abstract] OR "primary immune deficien*"[Title/Abstract] OR "inborn errors of immunity"[Title/Abstract]) AND ("screening"[Title/Abstract] OR "warning signs"[Title/Abstract] OR "algorithm"[Title/Abstract]))
- **Google Scholar:** ("primary immunodeficien*" OR "primary immune deficien*" OR "inborn errors of immunity") AND ("screening" OR "warning signs" OR "algorithm")

Search result

The following flow diagram summarizes the articles identified in the search:

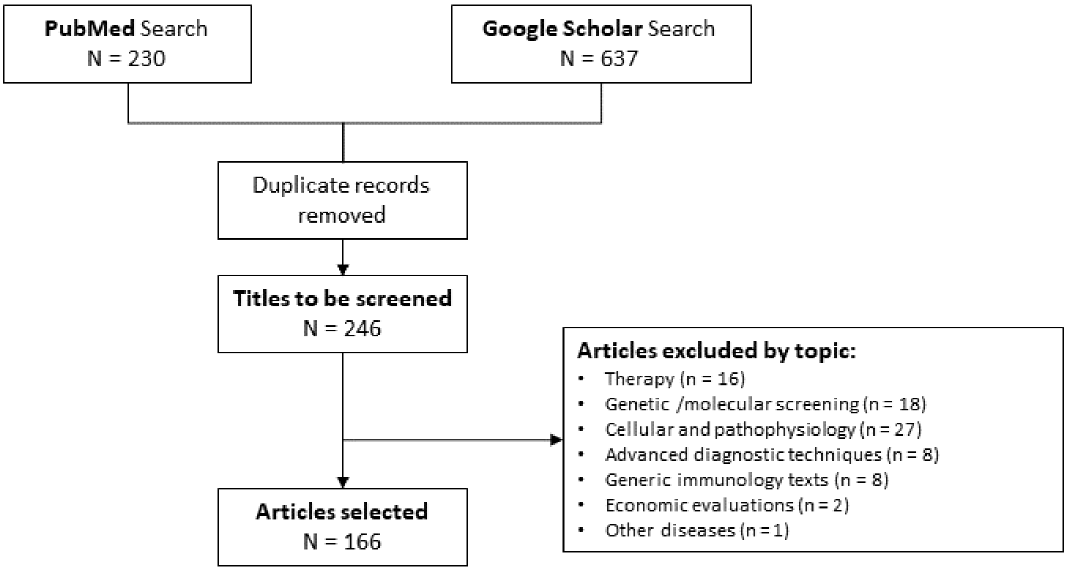

Table S1. Survey response distribution among healthcare providers

	**Answering the pediatric survey** (N=16)	**Answering the adult survey** (N=10)
Pediatric immunologist	11 (69%)	0
General (adult) immunologist	1 (6%)	9 (90%)
Primary care pediatrician	4 (25%)	0
Primary care physician	0	1 (10%)

Table S2. Survey results for pediatric warning signs

Warning Sign	Min Agreement	Max Agreement	Average Agreement	Standard Deviation	50th Percentile
≥ 6 acute otitis media/year in patients < 3 years of age; ≥ 4/year in patients ≥ 3 years of age; or ≥ 10 total	2	4	3.438	0.727	4
≥ 2 sinusitis or orbital cellulitis/year	2	4	3.500	0.632	4
≥ 2 pneumonias/year or ≥ 3 pneumonias/2 years	2	4	3.500	0.730	4
≥ 2 months of antibiotic treatment	2	4	3.250	0.683	3
Failure to thrive	1	4	3.250	0.931	4
Deep abscesses (in organs)	4	4	4.000	0.000	4
Abscesses; in lymph nodes or otherwise	2	4	3.313	0.793	4
Mucocutaneous candidiasis (oropharyngeal, cutaneous or vaginal); ≥ 1 in patients ≥ 6 months of age; ≥ 2 in patients < 6 months of age	2	4	2.750	0.856	3
≥ 2 systemic infections (including sepsis)	4	4	4.000	0.000	4
≥ 3 hospital admissions/year	2	4	3.063	0.854	3
≥ 1 serious infections that alone indicate IEI study (HSV meningitis, etc.)	4	4	4.000	0.000	4
Family history compatible with IEI manifestations (hematological malignancies, severe infections, etc.)	1	4	3.563	0.814	4
Consanguinity	2	4	3.250	0.775	3
Cytopenia; regardless of autoimmune or not	2	4	3.125	0.806	3
Systemic autoimmune diseases (celiac disease, arthritis, autoimmune hemolytic anemia, etc.)	2	4	3.063	0.680	3
Food intolerance/allergy	1	4	2.375	1.088	2
Endocrinopathology; Hypothyroidism, hyperparathyroidism, diabetes, etc. (Not described as autoimmune)	2	4	2.938	0.680	3
Hematological malignancy	2	4	3.188	0.834	3
Solid organ neoplasm	2	4	3.000	0.926	3
Presence of dental/palatal abnormalities	1	4	2.813	0.981	3
Chronic diarrhea; or ≥ 4 diarrhea-coding/year	2	4	3.375	0.619	3
Viral skin infection; or ≥ 3/year	1	4	3.125	1.025	4
Chronic eczema	2	4	3.000	0.894	3
Recurrent fever	3	4	3.813	0.403	4
Inflammatory bowel disease in patients ≥ 2 years of age	1	4	3.000	0.894	3
Inflammatory bowel disease in patients < 2 years of age	2	4	3.688	0.704	4
Bronchiectasis without cystic fibrosis	3	4	3.875	0.342	4
≥ 2 items of those previously described	1	4	2.938	1.063	3

Agreement refers to the degree of agreement or disagreement with the proposed items and their weighting, being 1: strongly disagree, 2: disagree; 3: agree and 4: strongly agree.
Data analyses from 16 survey respondents, 10 of whom are identified as pediatric immunologists, 2 identified as adult/mixed immunologists and 4 as primary care pediatricians.
HSV: Herpes Simplex Virus, IEI: Inborn errors of immunity

Table S3. Survey results for adult warning signs

Warning Sign	Min Agreement	Max Agreement	Average Agreement	Standard Deviation	50th Percentile
≥ 2 acute otitis media/year	2	4	2.900	0.738	3
≥ 2 severe sinusitis/year	3	4	3.300	0.483	3
≥ 2 pneumonias/10 years	1	4	2.800	1.135	3
Chronic diarrhea with weight loss	2	4	3.000	0.667	3
Deep abscesses (in organs and ganglia)	3	4	3.800	0.422	4
≥ 2 recurrent skin abscesses	1	4	2.700	1.059	3
Oropharyngeal or cutaneous candidiasis (excluding vaginal candidiasis)	1	4	2.900	0.994	3
Recurrent viral infections (colds, herpes, warts, condylomas, etc.); or ≥ 6 episodes/year	3	4	3.444	0.527	3
≥ 2 systemic infections (including sepsis)	3	4	3.900	0.316	4
≥ 3 hospital admissions/year	1	4	2.800	0.919	3
≥ 1 serious infections that alone indicate IEI study (HSV meningitis, etc.)	3	4	3.800	0.422	4
Infections with bacteria of the TB family but not very virulent (Atypical mycobacteria infection)	2	4	3.500	0.707	4
Family history compatible with IEI manifestations (hematological malignancies, severe infections, etc.)	2	4	3.200	0.919	4
Consanguinity	2	4	2.900	0.876	3
Cytopenia; regardless of autoimmune or not	2	4	2.700	0.675	3
Systemic autoimmune diseases (celiac disease, arthritis, autoimmune hemolytic anemia, etc.)	2	4	2.900	0.568	3
Hematological malignancy	1	4	2.800	0.789	3
Solid organ neoplasm	1	4	2.400	0.966	3
Recurrent fever	3	4	3.800	0.422	4
Inflammatory bowel disease	2	4	2.800	0.632	3
Bronchiectasis without cystic fibrosis	1	4	3.400	0.966	4
≥ 2 items of those previously described	2	4	3.100	0.876	3

Agreement refers to the degree of agreement or disagreement with the proposed items and their weighting, being 1: strongly disagree, 2: disagree; 3: agree and 4: strongly agree.
Data analyses from 10 survey respondents, 9 of whom are identified as adult immunologists, and 1 as a primary care physician.
HSV: Herpes Simplex Virus, IEI: Inborn errors of immunity, TB: Tuberculosis

List of ICD-10-CM codes associated with each warning sign

Pediatrics warning signs:

≥ 10 acute otitis media: H65 (and all subdiagnoses included in H65.0, H65.01, H65.02, H65.03 and H65.09); H66 (and all subdiagnoses); H67 (and all subdiagnoses); H73.0 (and all subdiagnoses); H73.1; H73.2; H92 (and all subdiagnoses).

≥ 3 sinusitis or orbital cellulitis: J01 (and all subdiagnoses); H05.01 (double scoring: one episode is enough, two episodes are not needed); J32 (double scoring: one episode is enough, two episodes are not needed).

≥ 3 pneumonias: J13; J14; J15 (and all subdiagnoses); J16; J17; J18 (and all subdiagnoses); J85.0 (one episode is enough); J85.1 (one episode is enough); J85.2 (one episode is enough); A37.01; A37.11; A37.81; A37.91.

Failure to thrive: R62.51; R62.52; R62.7; R63.4; R63.6; R64; P92.6.

Deep abscesses (in organs): A06.89; A17.81; A54.82; B43.1; D73.3; E32.1; G06 (and all subdiagnoses); G07; K63.0; K65.1; K68.1; K75.0; N15.1.

≥ 3 recurrent skin abscesses (internal note: 3 episodes separated by at least 30 days are required for scoring any of the codes except for L04, where only one episode is sufficient for scoring): K11.3; K12.2; K61; L02 (and all subdiagnoses; to score in this category, more than 2 episodes separated by 30 days are required); M65.0; M71.0; N34.0; N48.21; N70; N73; N76.4; H60.0; L04.

Mucocutaneous candidiasis (oropharynx, cutaneous, excluded vaginal) in patients ≥ 12 months of age: ≥ 2 episodes: B37.0; B37.2; B37.83; B37.84; B37.9.

≥ 2 systemic infections (including sepsis): J39.0; J39.1; H05.02; H05.03; H75.0; M72.8; M86 (and all subdiagnoses); M46.2 (and all subdiagnoses); M46.3 (and all subdiagnoses); M00 (and all subdiagnoses); M46.5 (and all subdiagnoses); M01 (and all subdiagnoses); M60.0 (and all subdiagnoses); M60.8 (and all subdiagnoses); M60.9; N49.3; G00 (and all subdiagnoses); G01 (and all subdiagnoses); G02 (and all subdiagnoses); G03 (and all subdiagnoses); G04 (and all subdiagnoses); G05 (and all subdiagnoses); G06 (and all subdiagnoses); G07 (and all subdiagnoses); J98.5 (and all subdiagnoses); J85.3; J86 (and all subdiagnoses); I40 (and all subdiagnoses); I41 (and all subdiagnoses); I30 (and all subdiagnoses); I32; I33; I38; A39 (and all subdiagnoses); A40 (and all subdiagnoses); A41 (and all subdiagnoses,); O85; P36 (and all subdiagnoses, except: P36.0 and P36.4); R65.2 (and all subdiagnoses); R78.81; A07.2; B06.8 (and all subdiagnoses); B06.0 (and all subdiagnoses); B05.0; B05.1; B05.2; B05.3; B05.4; B05.8; B01.0; D61.81.

≥ 1 serious infection that alone indicate IEI study (Meningitis caused by HSV, etc.): A02.22; A02.21; A02.1; A02.25; A02.23; A02.24; A32 (and all subdiagnoses); A43; B59; J84.2; B37.5; B37.1; B37.6; B37.7; B37.81; B37.82; B37.89; B38.3; B38.4; B38.7; B45.1; B45.2; B45.3; B45.7; B45.8; B45.9; B00.3; B00.4; B00.7; B00.82; B02.0; B02.1; B02.7; A07.2; B39.3; A07.3; B25.0; B25.8 (includes: cytomegaloviral encephalitis); B58.2; B58.3; B58.81; A81.2; A31.2; A17 Tu (and all subdiagnoses); A18 (and all subdiagnoses); A19 (and all subdiagnoses); A48.0; C46 (and all subdiagnoses); D59.3; D76.1; D76.2; D76.3; Certain disorders affecting the immune system (including all diagnoses and subdiagnoses of D80-D84); D89.82; D89.9; K13.3; E31.0; D70.4; E70.33; T78.3.

Family history of inborn errors of immunity: Z83.2.

Consanguinity or other family history compatible with manifestations of inborn errors of immunity (lymphomas, etc.): Z84.3; Z80.7.

Cytopenia (not specified as autoimmune): D53.9; D59.4; D59.9; D69.6; D70.3; D70.8; D70.9; D70; D72.1; D72.81 (and all subdiagnoses); D73.0; D75.9; D60.0; D60.8; D60.9; D61.89; D61.9; D61.3; D61.818; D61.82.

Autoimmune cytopenia: D59.1; D59.0; D69.3; D69.4 (and all subdiagnoses).

Systemic autoimmune diseases, not including autoimmune cytopenia (celiac disease, arthritis, etc): D86 (and all subdiagnoses); D89.0; D89.2; E06.3; E10 (and all subdiagnoses); K90.0; M05 (and all subdiagnoses); M06 (and all subdiagnoses); M08 (and all subdiagnoses); M13.0; M13.1 (and all subdiagnoses); M13.8 (and all subdiagnoses); M30 (and all subdiagnoses); M31 (and all subdiagnoses, except: M31.6); M79.3; M54.0; L93 (and all subdiagnoses); M35.6; M46.0 (and all subdiagnoses); M46.1; M46.4 (and all subdiagnoses); M46.8 (and all subdiagnoses); M46.9 (and all subdiagnoses); M32 (and all subdiagnoses); M33 (and all subdiagnoses); M34 (and all subdiagnoses); M45 (and all subdiagnoses); L63 (and all subdiagnoses); L80; E27.1; L40 (and all subdiagnoses); L52; E70.30; E70.39; M35.0 (and all subdiagnoses); M35.1; M35.2; M35.3; M35.8; M35.9.

Endocrinopathology: Hypothyroidism, hyperparathyroidism, diabetes, etc. (Not described as autoimmune): E08 (including all diagnoses); E16.9; E20 (and all subdiagnoses); E21 (and all subdiagnoses); E22 (and all subdiagnoses, except: E22.2); E23.0; E23.2; E24.8; E24.9; P71.1; P71.4; E03 (and all subdiagnoses); E04 (and all subdiagnoses, except: E4.1); E05 (and all subdiagnoses); E06.1; E06.2; E06.5; E06.9; E07 (including all diagnoses); E27.40; E27.5; E27.9; E25.8; E25.9; E26.0; E26.9; E28 (and all subdiagnoses, except: E28.0, E28.1, E28.2, E28.31 and its subdiagnoses); E29 (and all subdiagnoses); E31.1; E34 (and all subdiagnoses, except: E34.2).

Hematological malignancy: C81-C96 (and all subdiagnoses of C81 to C96, except: C84.0 and all subdiagnoses, C88.0, C88.2, C90.0 and all subdiagnoses); D46 (and all subdiagnoses).

Solid organ neoplasia (only those that have been associated with inborn errors of immunity in pediatrics: thyroid): C73-C75 (and all subdiagnoses C73 to C75); C7A (and all subdiagnoses); E31.2 (and all subdiagnoses); D44 (and all subdiagnoses); D43 (and all subdiagnoses).

Oral (dental/palatal) anomalies: Q35 (and all subdiagnoses); Q36 (and all subdiagnoses); Q37 (and all subdiagnoses); M26.06; M26.02; M26.04; K00.0; K00.4; K00.6; K05.11; K12.0; K13.0; K13.21.

Chronic diarrhea; or ≥ 10 episodes of acute diarrhea: K52.2; K52.8 (and all subdiagnoses); R19.7; A09; K52.9; A04 (and all subdiagnoses); A02.0; A03 (and all subdiagnoses); A07.1.

Chronic viral skin infection; or ≥ 20 acute episodes: B00.1; B00.0; B07 (and all subdiagnoses); B08 (and all subdiagnoses); B09.

Chronic eczema or other dermatological manifestations related to inborn errors of immunity: L20 (and all subdiagnoses); L50 (and all subdiagnoses); L83; L85.0; L98.2; L98.3.

Recurrent fever: A68.9; J03.91.

Inflammatory bowel disease in patients ≥ 2 years of age: K51 (and all subdiagnoses); K50 (and all subdiagnoses); K52.89.

Inflammatory bowel disease in patients < 2 years of age: K51 (and all subdiagnoses); K50 (and all subdiagnoses); K52.89.

Bronchiectasis without cystic fibrosis: J47 (and all subdiagnoses) exclude if presence of cystic fibrosis (E84 and subdiagnoses).

Vaccine reaction: T50.B95; T50.A95; T50.A25.

Exclusions from the scoring system if coded: B20 Human immunodeficiency virus [HIV] disease; Z21 Asymptomatic status of human immunodeficiency virus [HIV] infection.

Corresponding diagnostic codes ICD10-CM (adults):

≥ 8 acute otitis media: H65 (and all subdiagnoses included in H65.0, H65.0, H65.02, H65.03 and H65.09); H66 (and all subdiagnoses); H67 (and all subdiagnoses). H73.0 (and all subdiagnoses). H73.1; H73.2; H92 (and all subdiagnoses).

≥ 8 sinusitis or chronic sinusitis: J01 (and all subdiagnoses); H05.01 (double scoring: one episode is enough, two episodes are not needed); J32 (double scoring: one episode is enough, two episodes are not needed).

≥ 3 pneumonias: J13; J14; J15 (and all subdiagnoses); J16; J17; J18 (and all subdiagnoses); J85.0 (one episode is enough); J85.1 (one episode is enough); J85.2 (one episode is enough); A37.01; A37.11; A37.81; A37.91.

Chronic diarrhea (coding of 10 or more episodes of the following items): R63.4; R64; R62 (and all subdiagnoses); K52.2; K52.8 (and all subdiagnoses); R19.7; A09; K52.9; A04 (and all subdiagnoses); A02.0; A03 (and all subdiagnoses); A07.1.

Deep abscesses (in organs and/or ganglia): A06.89; A17.81; A54.82; B43.1; D73.3; E32.1; G06 (and all subdiagnoses); G07; K63.0; K65.1; K68.1; K75.0; N15.1; L04.

Recurrent skin abscesses of repetition (3 or more): K11.3; K12.2; K61; L02 (and all subdiagnoses); M65.0; M71.0; N34.0; N41.2; N48.21; N70; N71; N73 (except N73.6); N76.4; H60.0.

Oropharyngeal or cutaneous candidiasis (excluding vaginal candidiasis): B37.0; B37.2; B37.83; B37.84; B37.9; B37.81.

Recurrent viral infections (colds, herpes, warts, condylomas, etc.) 25 or more episodes: B00.1; B00.0; B00.9; B01.9; B01.2; B01.8; B02.9; B02.3; B05 (and all subdiagnoses); B06.9; B07 (and all subdiagnoses); B08 (and all subdiagnoses); B09; B10.8 (and all subdiagnoses); B15 (and all subdiagnoses); B17.2; B25.1; B26.9; B27 (and all subdiagnoses); B30 (and all subdiagnoses); B33.8; B34 (and all subdiagnoses); A08 (and all subdiagnoses); A09; J00 (and all subdiagnoses); J02.9; J04 (and all subdiagnoses); J05 (and all subdiagnoses); J06 (and all subdiagnoses); J09 (and all subdiagnoses); J09 (and all subdiagnoses); J09 (and all subdiagnoses); J10 (and all subdiagnoses); J11 (and all subdiagnoses); J12 (and all subdiagnoses); J20 (and all subdiagnoses); J21 (and all subdiagnoses); J40.

2 or more systemic infections including sepsis: J39.0; J39.1; H05.02; H05.03; H75.0; M72.8; M86 (and all subdiagnoses); M46.2 (and all subdiagnoses); M46.3 (and all subdiagnoses); M00 (and all subdiagnoses); M46.5 (and all subdiagnoses); M01 (and all subdiagnoses); M60.0 (and all subdiagnoses); M60.8 (and all subdiagnoses); M60.9; N49.3; G00 (and all subdiagnoses); G01 (and all subdiagnoses); G02 (and all subdiagnoses); G03 (and all subdiagnoses); G04 (and all subdiagnoses); G05 (and all subdiagnoses); G06 (and all subdiagnoses); G07 (and all subdiagnoses); J98.5; J85.3; J86 (and all subdiagnoses); I40 (and all subdiagnoses); I41 (and all subdiagnoses); I30 (and all subdiagnoses); I32; I33; I38; A39 (and all subdiagnoses); A40 (and all subdiagnoses); A41 (and all subdiagnoses); O85; R65.2 (and all subdiagnoses); R78.81; A07.2; B06.8 (and all subdiagnoses); B06.0 (and all subdiagnoses); B05.0; B05.1; B05.2; B05.4; B05.8; B01.0; D61.81.

Unique severe condition that alone require study for inborn errors of immunity: A02.22; A02.21; A02.1; A02.25; A02.23; A02.24; A32 (and all subdiagnoses); A43; B59; J84.2; B37.5; B37.1; B37.6; B37.7; B37.82; B37.89; B38.3; B38.4; B38.7; B45.1; B45.2; B45.3; B45.7; B45.8; B45.9; B00.3; B00.4; B00.7; B00.82; B02.0; B02.1; B02.7; A07.2; B39.3; A07.3; B25.0; B25.8 (includes: cytomegaloviral encephalitis); B58.2; B58.3; B58.81; A81.2; A31.2; A17 (and all subdiagnoses); A18 (and all subdiagnoses); A19 (and all subdiagnoses); A48.0; C46 (and all subdiagnoses); D59.3; D76.1; D76.2; D76.3; (including all diagnoses and all subdiagnoses of D80-D84); D89.82; D89.9; K13.3; E31.0; D70.4; E70.33.

Atypical mycobacteria infection: A31.0; A31.1; A31.8; A31.9; A30.

Family history of inborn errors of immunity (hematological neoplasms, serious infections, etc.): Z83.2.

Consanguinity or other family history compatible with manifestations of inborn errors of immunity (hematological neoplasms): Z84.3; Z80.7

Presence of cytopenia (without specifying if autoimmune): D53.9; D59.4; D59.9; D69.6; D70.3; D70.8; D70.9; D70; D72.1; D72.81 (and all subdiagnoses); D73.0; D75.9; D60.0; D60.8; D60.9; D61.89; D61.9; D61.3; D61.818; D61.82.

Presence of bronchiectasis without cystic fibrosis: J47 (and all subdiagnoses) exclude if presence of cystic fibrosis (E84 and subdiagnoses).

Autoimmune cytopenia: D59.1; D59.0; D69.3; D69.4 (and all subdiagnoses).

Systemic and endocrine autoimmune diseases (celiac disease, arthritis, systemic lupus, thyroiditis, etc.): D86 (and all subdiagnoses); D89.0; D89.1; D89.2; E06.3; E10 (and all subdiagnoses); K90.0; M05 (and all subdiagnoses); M06 (and all subdiagnoses); M08 (and all subdiagnoses); M13.0; M13.1 (and all subdiagnoses); M13.8 (and all subdiagnoses); M30 (and all subdiagnoses); M31 (and all subdiagnoses); M79.3; M54.0; L93 (and all subdiagnoses); M35.6; M46.0 (and all subdiagnoses); M46.1; M46.4 (and all subdiagnoses); M46.8 (and all subdiagnoses); M46.9 (and all subdiagnoses); M32 (and all subdiagnoses); M33 (and all subdiagnoses); M34 (and all subdiagnoses); M35 (and all subdiagnoses); M45 (and all subdiagnoses); L63 (and all subdiagnoses); L80; E27.1; L40 (and all subdiagnoses); L52; E70.30; E70.39; M35.0 (and all subdiagnoses); M35.1; M35.2; M35.3; M35.8; M35.9; E20 (and all subdiagnoses); E21 (and all subdiagnoses); E21 (and all subdiagnoses); E03 (and all subdiagnoses); E04 (and all subdiagnoses); E05 (and all subdiagnoses); E06.1; E06.2; E06.5; E06.9; E07 (including all diagnoses); E31.1.

Hematological neoplasia (excluding multiple myeloma, chronic myeloid leukemia, Waldenström's disease, etc): C81 (and all subdiagnoses); C82 (and all subdiagnoses); C83 (and all subdiagnoses); C84 (and all subdiagnoses); C85 (and all subdiagnoses); C86 (and all subdiagnoses); C88.3; C88.4; C88.8; C88.9; C90.1 (including all diagnoses); C91.0 (including all diagnoses); C91.3 (including all diagnoses); C91.6 (including all diagnoses); C91.A (including all diagnoses); C91.9 (including all diagnoses); C92.0 (including all diagnoses); C92.3 (including all diagnoses); C92.4 (including all diagnoses); C92.5 (including all diagnoses); C92.6 (including all diagnoses); C92.A (including all diagnoses); C92.Z (including all diagnoses); C92.9 (including all diagnoses); C93.0 (including all diagnoses); C93.3 (including all diagnoses); C93.Z

(including all diagnoses); C93.9 (including all diagnoses); C94.0 (including all diagnoses); C94.2 (including all diagnoses); C94.3 (including all diagnoses); C94.8 (including all diagnoses); C95.0 (including all diagnoses); C95.9 (including all diagnoses); C96 (including all diagnoses); D46 (and all subdiagnoses); D47.Z (and all subdiagnoses); D47.9 (and all subdiagnoses); D47.0 (and all subdiagnoses).

Solid organ neoplasia (only those related with inborn errors of immunity: skin, stomach, thyroid): C16 (and all subdiagnoses); C43-C44 (and all subdiagnoses from C43 to C44); C45 (and all subdiagnoses); C73-C75 (and all subdiagnoses C73 to C75); C7A (and all subdiagnoses); E31.2 (and all subdiagnoses); D44 (and all subdiagnoses); D00.2.

Inflammatory bowel disease: K51 (and all subdiagnoses); K50 (and all subdiagnoses).

Oral (dental/palatal) anomalies: Q35 (and all subdiagnoses); Q36 (and all subdiagnoses); Q37 (and all subdiagnoses); M26.06; M26.02; M26.04; K00.0; K00.4; K00.6; K05.3; K05.11; K12.0; K13.0; K13.21.

Chronic eczema or other dermatological manifestations related to inborn errors of immunity: L20 (and all subdiagnoses); L50 (and all subdiagnoses); L83; L85.0; L98.3.

Recurrent fever: A68.9; J03.91.

Exclusions from the scoring system if coded: B20 Human immunodeficiency virus [HIV] disease; Z21 Asymptomatic status of human immunodeficiency virus [HIV] infection.

Table S4. Main demographic characteristics of patients included in the retrospective testing of the scoring system in a cohort of patients with inborn errors of immunity. No. (%)

**Age Group**	**Overall** (N = 305)	**Male** (N = 149)	**Female** (N = 156)
Age 0 to 1 years	8 (2.6)	4 (2.7)	4 (2.6)
Age 2 to 4 years	23 (7.5)	17 (11.4)	6 (3.8)
Age 5 to 9 years	52 (17)	27 (18.1)	25 (16)
Age 10 to 14 years	51 (16.7)	25 (16.8)	26 (16.7)
Age 15 to 19 years	33 (10.8)	20 (13.4)	13 (8.3)
Age 20 to 24 years	23 (7.5)	13 (8.7)	10 (6.4)
Age 25 to 29 years	13 (4.3)	10 (6.7)	3 (1.9)
Age 30 to 34 years	11 (3.6)	7 (4.7)	4 (2.6)
Age 35 to 39 years	11 (3.6)	4 (2.7)	7 (4.5)
Age 40 to 44 years	9 (3)	1 (0.7)	8 (5.1)
Age 45 to 49 years	11 (3.6)	6 (4)	5 (3.2)
Age 50 to 54 years	6 (2)	1 (0.7)	5 (3.2)
Age 55 to 59 years	14 (4.6)	3 (2)	11 (7.1)
Age 60 to 64 years	12 (3.9)	4 (2.7)	8 (5.1)
Age 65 to 69 years	9 (3)	2 (1.3)	7 (4.5)
Age 70 to 74 years	8 (2.6)	3 (2)	5 (3.2)
Age 75 to 79 years	8 (2.6)	1 (0.7)	7 (4.5)
Age 80 to 84 years	1 (0.3)	1 (0.7)	0 (0)
Age 85 to 89 years	1 (0.3)	0 (0)	1 (0.6)
Age 90 to 94 years	1 (0.3)	0 (0)	1 (0.6)
Age 95 years or more	0 (0)	0 (0)	0 (0)

Table S5. International Union of Immunological Societies (IUIS) diagnosis group classification of pediatric patients included in the retrospective testing of the scoring system in a cohort of patients with inborn errors of immunity.

**IUIS group classification**	**High risk** (N = 60)	**Medium risk** (N = 20)	**Low risk** (N = 104)	**Total** (N = 184)
Combined immunodeficiencies	3	3	12	18
Combined immunodeficiencies with associated or syndromic features	7	2	18	27
Predominantly antibody deficiencies	9	4	19	32
Diseases of immune dysregulation	7	0	11	18
Congenital defects of phagocyte number, function, or both	6	2	8	16
Defects in innate immunity	3	3	3	9
Autoinflammatory disorders	17	3	31	51
Complement deficiencies	6	3	2	11
Bone marrow failure	2	0	0	2

Table S6. International Union of Immunological Societies (IUIS) diagnosis group classification of adult patients included in the retrospective testing of the scoring system in a cohort of patients with inborn errors of immunity.

**IUIS group classification**	**High risk** (N = 36)	**Medium risk** (N = 13)	**Low risk** (N = 72)	**Total** (N = 121)
Combined immunodeficiencies with associated or syndromic features	0	2	3	5
Predominantly antibody deficiencies	32	11	66	109
Diseases of immune dysregulation	1	0	1	2
Congenital defects of phagocyte number, function, or both	3	0	2	5

Table S7. Main demographic characteristics of the source population for the pilot implementation. No. (%)

**Age Group**	**Overall** (N = 19,391)	**Male** (N = 9,336)	**Female** (N = 10,055)
Age 0 to 1 years	320 (1.7)	151 (1.6)	169 (1.7)
Age 2 to 4 years	448 (2.3)	239 (2.6)	209 (2.1)
Age 5 to 9 years	902 (4.7)	473 (5.1)	429 (4.3)
Age 10 to 14 years	927 (4.8)	472 (5.1)	455 (4.5)
Age 15 to 19 years	824 (4.2)	430 (4.6)	394 (3.9)
Age 20 to 24 years	964 (5)	489 (5.2)	475 (4.7)
Age 25 to 29 years	1114 (5.7)	531 (5.7)	583 (5.8)
Age 30 to 34 years	1270 (6.5)	634 (6.8)	636 (6.3)
Age 35 to 39 years	1494 (7.7)	747 (8)	747 (7.4)
Age 40 to 44 years	1732 (8.9)	906 (9.7)	826 (8.2)
Age 45 to 49 years	1562 (8.1)	757 (8.1)	805 (8)
Age 50 to 54 years	1391 (7.2)	672 (7.2)	719 (7.2)
Age 55 to 59 years	1187 (6.1)	593 (6.4)	594 (5.9)
Age 60 to 64 years	1150 (5.9)	550 (5.9)	600 (6)
Age 65 to 69 years	1080 (5.6)	524 (5.6)	556 (5.5)
Age 70 to 74 years	1033 (5.3)	453 (4.9)	580 (5.8)
Age 75 to 79 years	821 (4.2)	334 (3.6)	487 (4.8)
Age 80 to 84 years	711 (3.7)	311 (3.3)	400 (4)
Age 85 to 89 years	471 (2.4)	155 (1.7)	316 (3.1)
Age 90 to 94 years	243 (1.3)	68 (0.7)	175 (1.7)
Age 95 years or more	58 (0.3)	8 (0.1)	50 (0.5)
